# An appraisal of clinical practice guidelines for the appropriate use of echocardiography for adult infective endocarditis—the timing and mode of assessment (TTE or TEE)

**DOI:** 10.1186/s12879-021-05785-6

**Published:** 2021-01-21

**Authors:** Peihan Xie, Xiaodong Zhuang, Menghui Liu, Shaozhao Zhang, Jia Liu, Donghong Liu, Xinxue Liao

**Affiliations:** 1 Department of Cardiology, The First Affiliated Hospital of Sun Yat-Sen University; NHC Key Laboratory of Assisted Circulation (Sun Yat-sen University), 58 Zhongshan 2nd Rd, Guangzhou, 510080 People’s Republic of China; 2 Department of Ultrasonography, The First Affiliated Hospital of Sun Yat-Sen University, Guangzhou, People’s Republic of China

**Keywords:** Infective endocarditis, Guideline, Transesophageal echocardiography, Transthoracic echocardiography

## Abstract

**Background:**

Echocardiography (echo) is the primary imaging modality for infective endocarditis (IE). However, the recommendations on timing and mode selection for transesophageal echocardiography (TEE) and transthoracic echocardiography (TTE) vary across guidelines, which can be confusing for clinical decision makers. In this case, we aim to appraise the quality of recommendations by appraising the quality of various guidelines.

**Methods:**

A search of guidelines containing recommendations for the appropriate use of echo in adult IE patients published in English between 2007 and 2019 was conducted. The APPRAISAL OF GUIDELINES FOR RESEARCH & EVALUATION II (AGREE II) instrument was applied independently by two reviewers to assess the integrated quality of the identified guidelines. The recommendations of concern are extracted from related chapters.

**Results:**

A total of 9 guidelines met the criteria, with AGREE II scores ranging from 36 to 79%, and the domain of “stakeholder involvement” received the lowest score. The most contentious issue is whether a follow-up TEE is mandatory in uncomplicated native valve IE with an initial positive TTE. Conflicting recommendations are presented with a low evidence level based on little evidence.

**Conclusions:**

In general, the recommendations proposed in the 9 identified guidelines on the appropriate use of echo are satisfying. The guideline quality score can be taken into account by the clinicians when evaluating the recommendations for clinical decisions. Additional studies with high evidence level should be conducted on the most controversial issues of whether a subsequent TEE is mandatory in uncomplicated native valve IE with an initial positive TTE.

**Supplementary Information:**

The online version contains supplementary material available at 10.1186/s12879-021-05785-6.

## Background

Echocardiography (echo) is the primary imaging modality for infective endocarditis (IE). A positive echo is defined by vegetation or abscess, and new dehiscence of the prosthetic valve is included as a major modified Duke criterion along with positive microorganisms [1]. In addition, echocardiographic examination is also able to show haemodynamic severity of the valve lesion [2]. The appropriate use of echo should be cost-effective, time-efficient and focused on potential associated complications [3, 4]. Timely and accurate guidance should be available for the diagnosis and management of IE. As per a single-center study, a considerable number of transesophageal echocardiography (TEE) exams were rated as inappropriately used for IE [5]. Over the past 15 years, there have been 9 guidelines that cover IE diagnosis and contain evidence-based recommendations on the appropriate use of echo (all published in English, and the latest version of each guideline was considered). However, these guidelines differ in their recommendations on TEE and transthoracic echocardiography (TTE) application in certain clinical scenarios, which can be confusing for clinical decision makers. APPRAISAL OF GUIDELINES FOR RESEARCH & EVALUATION (AGREE) is an instrument for comprehensive guideline evaluation across 6 domains, including scope and purpose, stakeholder involvement, rigor of development, clarity of presentation, applicability and editorial independence. AGREE II, the updated version developed in 2013, updated certain items from the original version [6]. A systematic review of the appropriate use of echo based on critical assessment and the quality comparison of different guidelines was presented to allow clinicians to make better decisions in certain confusing clinical scenarios.

## Methods

### Search process

A literature search of current clinical practice guidelines that contain recommendations on IE imaging was conducted on PubMed, Embase, Web of Science and websites of guideline development societies (websites of organizations and societies are shown in Table S1); “adult” “infective endocarditis”, “echocardiography”, “transesophageal echocardiography” and “transthoracic echocardiography” were searched either singly or in combination (the detailed search strategy used is shown in Table S2). The search covered the guidelines published in English from 5 June 2007 to 5 July 2019.

### Inclusion criteria

Guidelines that met the following criteria were included:

1. Met the definition of a guideline given by The Institution of Medicine [7], which was described as “systematically developed statements to assist practitioner and patient decisions on appropriate healthcare for specific clinical circumstance”.
2. Contained recommendations on the appropriate use of echo in the diagnosis, treatment, or follow-up process of IE.
3. Targeted adult patients.
4. Was the latest version with updated guidelines.
5. Was free to access the full text.
6. Was written in English, including translated versions of other languages.

Titles and abstracts were reviewed by 2 reviewers independently. After that, any disagreements were discussed and adjudicated through consensus with a third party. Finally, the final selection of guidelines was performed by the 2 reviewers together.

### Guideline appraisal and recommendation extraction

A comprehensive evaluation was performed on the 9 selected guidelines across the 6 domains of the AGREE II instrument: i) scope and purpose, ii) stakeholder involvement, iii) rigor of development, iv) clarity of presentation, v) applicability, and vi) editorial independence. Two reviewers evaluated 23 items across 6 domains independently by providing scores ranging from 1 to 7, where 1 represents strong disagreement due to no relevant information given or the concept being barely addressed, and 7 represents strong agreement due to exceptional reporting quality or fully meeting the criteria of AGREE II. The final score for each domain was obtained by the calculation formula given by AGREE II: the scaled domain score = (obtained score – minimum possible score)/(maximum possible score – minimum possible score). If the difference between the scores given by both reviewers for a certain item was greater than 20% of the lower score, a third reviewer reviewed and evaluated the guidelines [8]. The guidelines with an average score higher than 60% were marked as “Strongly recommended”, those with scores ranging from 30 to 60% were marked as “Recommended with some modification”, and those with scores lower than 30% were marked as “Not recommended” [9].

Except for the guidelines of the Chinese Society of Cardiology, version 2015 and the Swedish Society of Infectious Diseases, version 2007 (CSC 2015, SSID 2007) without the report of Conflicts of Interest (COI) [10, 11], we calculated the proportion of panel members with an industrial relationship (RWI) with authors reported in other guidelines and analyzed the correlation between the RWI ratio and AGREE II score using SPSS 25.0. Statistical significance was indicated when α<0.05 [8].

All recommendations on the appropriate use of echo, including the timing and mode (TTE and TEE), were extracted from the relevant chapters. In an effort to avoid phrasing confusion, the recommendation class and level of evidence quoted from SSID 2007 were converted into a unified form that is consistent with those used in other guidelines [11]. The recommendation grade and level of evidence were coded as I/II/III and A/B/C, respectively.

## Results

### Guidelines that meet the criteria

**Fig. 1:**
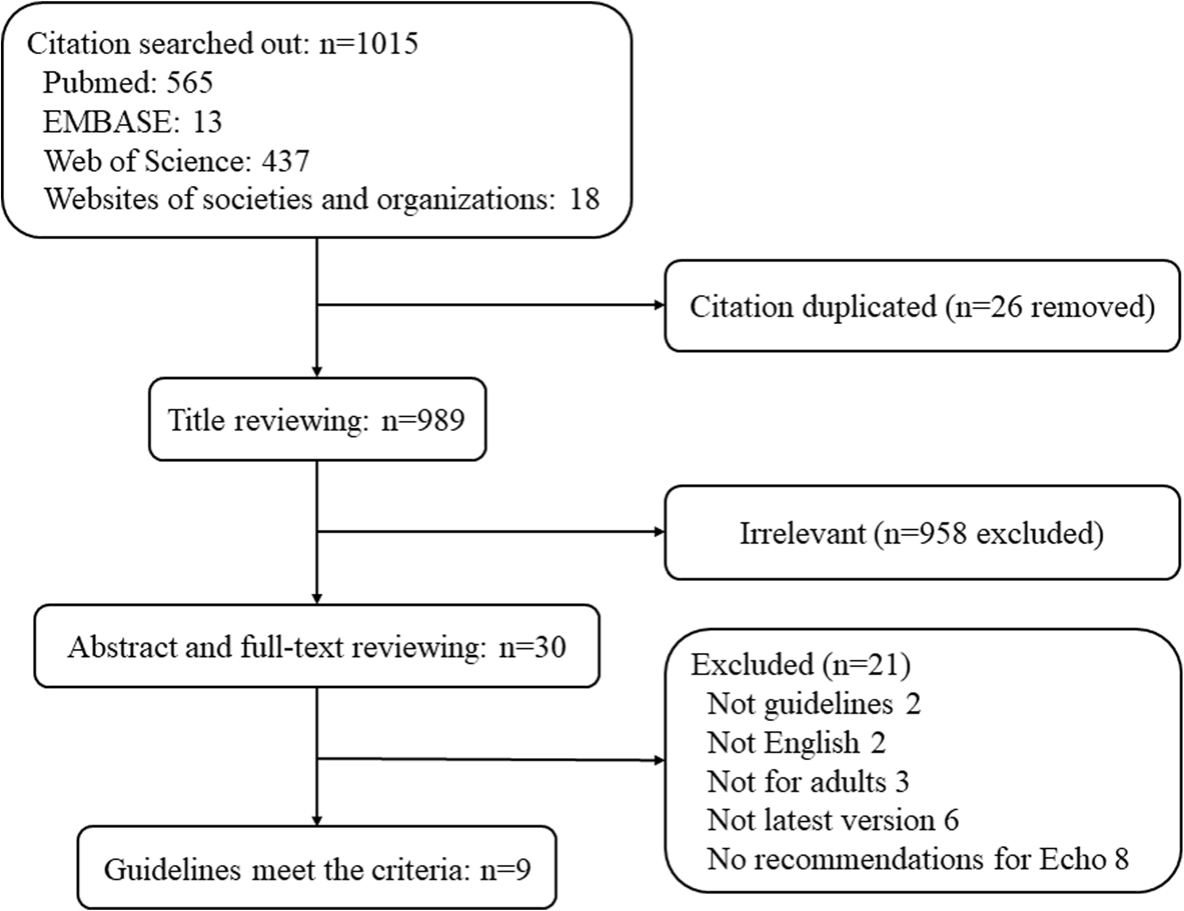
Flow diagram of inclusion/exclusion processes for the guidelines

A total of 1015 records were searched during the literature search, and 1006 records were removed after the review of the title, abstract, and full text (Fig. 1). As the outcome, 9 guidelines reported by 8 host organizations (some of them were joint endeavors) met the including criteria: National Heart Association of Malaysia, version 2017 (NHAM 2017); American Heart Association, version 2015 (AHA 2015); European Society of Cardiology, version 2015 (ESC 2015); British Society for Antimicrobial Chemotherapy, British Heart Rhythm Society, version 2014, (BSAC/BHRS 2014); Japanese Circulation Society, version 2017, (JCS 2017); British Society for Antimicrobial Chemotherapy, version 2011 (BSAC 2011); Spanish Society of Infectious Diseases and Clinical Microbiology, version 2015 (SEIMC 2015); CSC 2015; and SSID 2007 [10–18]. The guidelines are shown in Table S3. The guideline identifiers, host organizations, regions, average AGREE II scores, COI, number of echo recommendations and RWIs are summarized in Table 1.

**Table 1:** General characteristics of the included 9 guidelines

Guidelines identifier, Year^a^	Organization(s) responsible for guidelines development	Target population	Proportion of authors RWI	AGREE II score, %	Number of Echo recommendations	Guideline status
AHA, 2015	American Heart Association	Adult IE	4/16 (25.0%)	77	5	Strongly recommended
ESC, 2015	European Society of Cardiology	IE	13/20 (65.0%)	75	10	Strongly recommended
BSAC, 2011	British Society for Antimicrobial Chemotherapy	Adult IE	2/9 (22.2%)	54	7	Recommended
BSAC/BHRS, 2014	British Society for Antimicrobial Chemotherapy, British Heart Rhythm Society	Infections related to ICED	4/12 (33.3%)	62	5	Strongly recommended
SEIMC, 2015	Spanish Society of Infectious Diseases and Clinical Microbiology	IE caused by S.aureus	6/17 (35.3%)	53	5	Recommended
SSID, 2007	Swedish Society of Infectious Diseases	IE	–	36	6	Recommended
CSC, 2015	Chinese Society of Cardiology	Adult IE	–	39	9	Recommended
JCS, 2017	Japanese Circulation Society	IE	7/24 (29.2%)	55	10	Recommended
NHAM, 2017	National Heart Association of Malaysia	IE	0/13 (0%)	79	7	Strongly recommended

*IE* infective endocarditis, *ICED* implantable cardiac electronic device, *RWI* the proportion of panel members with an industry relationship, *AGREE II* APPRAISAL OF GUIDELINES FOR RESEARCH & EVALUATION II

^a^ The guideline references were listed in Table S4

### Guideline appraisal by AGREE II

**Fig. 2:**
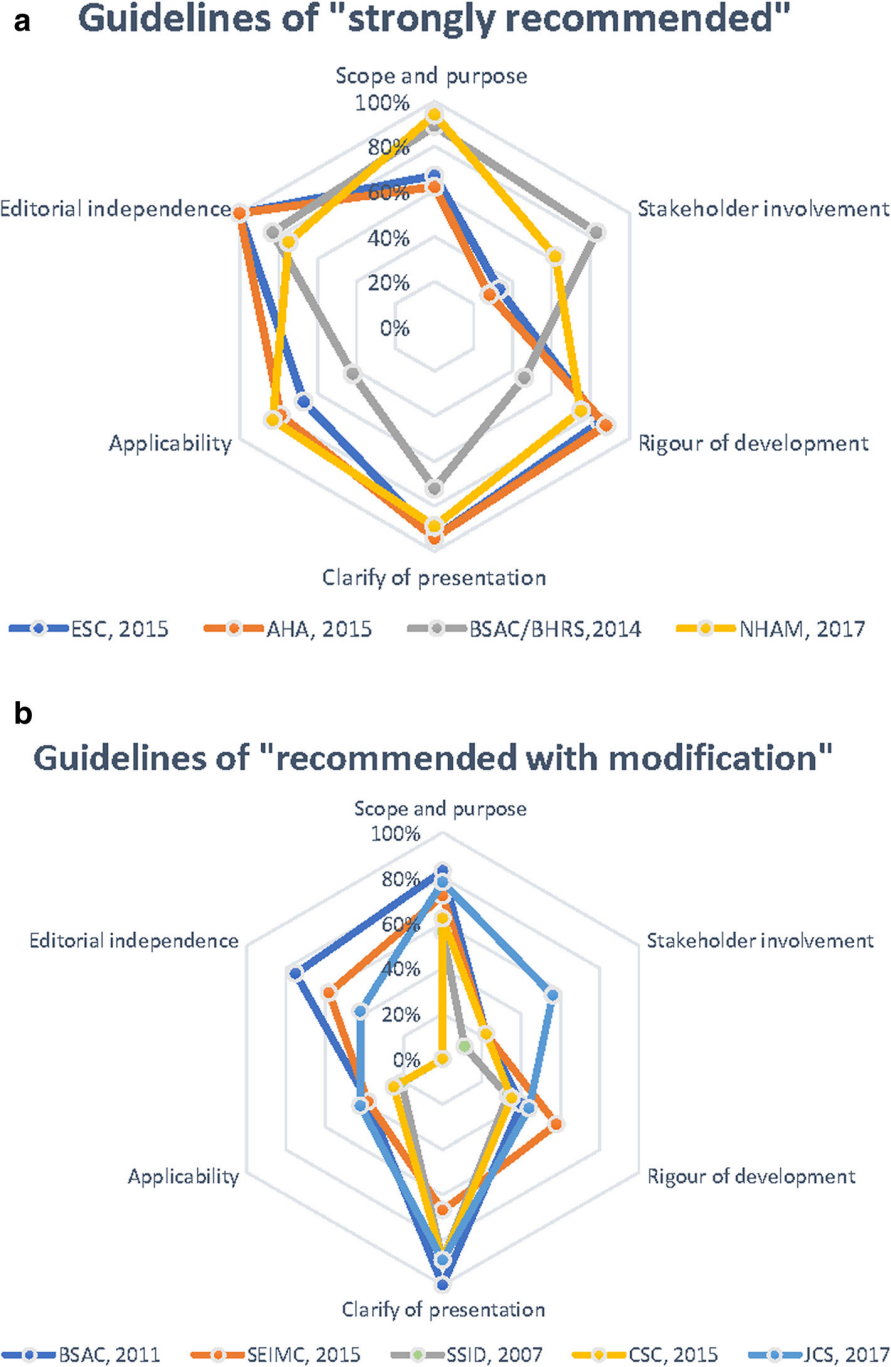
Rader charts of the AGREE II score distribution across 6 domains for the guidelines. The guidelines were divided into different charts by scale of aggregate score. AGREE II, appraisal of guidelines for research & evaluation. **a** Guidelines classified as “Strongly recommended”. **b** Guidelines classified as “Recommended with modification”

The scores of each guideline are presented in the radar charts (Fig. 2) and Table S4. The AGREE II scores for all guidelines ranged from 36 to 79%, with a median of 55%; among them, 4 guidelines with scores higher than 60% (NHAM 2017, AHA 2015, ESC 2015, and BSAC/BHRS 2014) [12–15] were rated as “Strongly recommended”, while the others, with scores ranging from 36 to 55%, (JCS 2017, BSAC 2011, SEIMC 2015, CSC 2015, and SSID 2007) [10, 11, 16–18] were rated as “Recommended with some modifications”. No guideline had a score lower than 30% or rated as “Not recommended”. There was no item with a greater than 20% difference between the scores given by the two reviewers.

Domain1 (scope and purpose) includes items on the overall goal, the specific health issues contained in the guideline, and its target population. In contrast with other domains, guideline performance in this domain is nonspecific; that is, most of them generally summarized the concerning issues without any further elaboration. NHAM 2017, BSAC/BHRS 2014, and BSAC 2011 [12, 15, 17] specifically expounded on the items following the AGREE II rules, thus receiving relatively high scores.

Domain2 (stakeholder involvement) concerns whether the guideline was developed by appropriate stakeholders and whether the views of its intended users were considered. This domain received the lowest average score, with two-thirds of the guidelines were scoring no more than 33%. Only NHAM 2017, BSAC/BHRS 2014, and JCS 2017 [12, 15, 16] specified the department to which the clinical guideline applied for the specialists. In addition, patients as the target population was reported only by BSAC/BHRS 2014 [15] in the external review.

Domain 3 (rigor of development) comprises the processes of evidence synthesis, the formulation of the recommendations and update procedures. CSC 2015 and SSID 2007 [10, 11] reported no information on the selection of evidence, while BSAC/BHRS 2014, JCS 2017 and BSAC 2011 [15–17] provided little description of this subject. In addition, only AHA 2015, ESC 2015 [13, 14] and NHAM 2017 [12] privided updated statements or detailed information on external expert reviews. The narrative of methods for formulating the recommendations were relatively clear, resulting in high scores.

Domain 4 (clarity of presentation) involves the clarity of the description and the format of the guideline. The guidelines received the highest average scores in this domain, and the variation in the scores was the smallest among all domains.

Domain 5 (applicability) addresses practical implementation, efforts for improving uptake, and resource implications. Additional disseminating materials were provided by only NHAM 2017 ESC 2015 and AHA 2015 [12–14]. Few guidelines mentioned cost implications. However, the monitoring and auditing criteria were set precisely by all guidelines, with an average score higher than 80%.

Domain 6 (editorial independence) pertains to transparency declarations, including the funding body and competing interests of the members of the guideline-development group. Nor information on the funding body or its influence on the content was mentioned by JCS 2017, CSC 2015 or SSID 2007 [10, 11, 16]. In addition, no records on the disclosure of potential COI were mentioned by SSID 2007 [11] or CSC 2015 [10]. As a result, there was no correlation between RWI and AGGEE II score (Pearson’s correlation *r* = − 0.081 *P* = 0.863) for the rest of the guidelines.

**Fig. 3:**
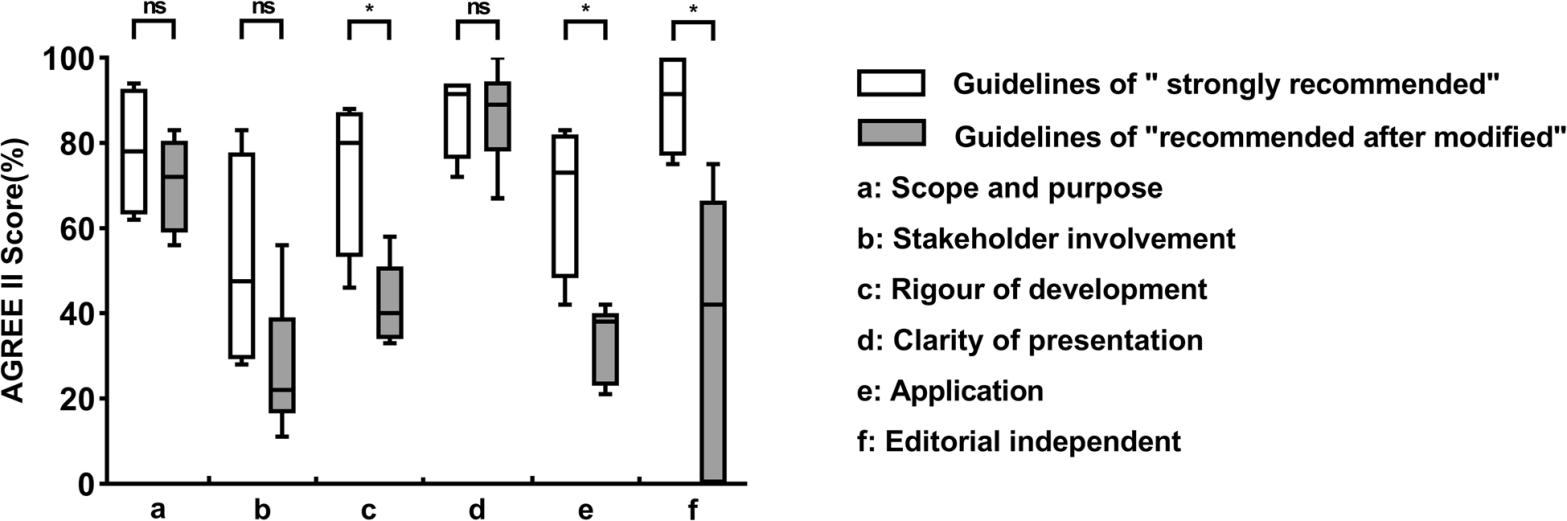
Box plot of the AGREE II score distribution comparison between “guidelines strongly recommended” and “guidelines recommended with modification” in different domains. ns: no significant difference. *: significant difference (*P* < 0.05)

More than half of the guidelines (5 out of 9) received scores lower than 60%. According to the independent-sample t-test, t’-test (editorial independence) and Wilcoxon rank-sum test (clarification of presentation), the guidelines classified as “Recommended with modification” were significantly different from those classified as “Strongly recommended” in the domains of “rigor of development” (*P* = 0.015), “applicability” (*P* = 0.005) and “editorial independence” (*P* = 0.024) (Fig. 3). With regard to specific items, 8 of the 9 guidelines received a score of zero on the “target population” item, which indicates that the guidelines generally failed to consider the view of patients in the recommendations.

### Recommendations on appropriate use of echo

The recommendations for various clinical scenarios were organized and listed in Table S5 (recommendations with controversies) and Table S6 (recommendations without controversies). As shown in the tables, both consensus and controversy were present, and the consensus outweighed the controversy.

Seven of the 9 guidelines included recommendations on the first-line modality of suspected IE. Except for the contradictory and inconclusive statement of SSID 2007, all other guidelines (6 out of 7) agreed that TTE should be the first choice (class of recommendation: I, level of evidence: B-C).

Five of the 9 guidelines recommended further TEE, when first-line TTE is proven to be nondiagnostic due to its poor echocardiographic window, due to the higher sensitivity of TEE (class of recommendation: I, level of evidence: B-C) [1].

For patients with suspected IE under a prosthetic heart valve/intracardiac device, TEE was recommended by 5 of the 9 guidelines (class of recommendation: I, level of evidence: B-C). Furthermore, suggestions for patients with implantable cardiac electronic devices (ICEDs) were given by the guidelines of BSAC/BHRS 2014, recommending that echo should be conducted for patients with implantable cardiac electronic device lead infection (LCED-LI), implantable cardiac electronic device-associated native or prosthetic valve endocarditis (LCED-IE) and suspected generator pocked infection concurrent with ICED-LI or ICED-IE (level of evidence: B-C) [15].

Echo is recommended for patients with *S. aureus* bacteremia (SAB) by 7 of the 9 guidelines (class of recommendation: IIa, level of evidence: B-C).

Follow-up echo (no mode was specified) was advised by 5 of the 9 guidelines after the onset of a suspected IE (class of recommendation: I, level of evidence: B-C). Among them, ESC 2015 and CSC 2015 further specified the clinical manifestations of a complication include new murmur, embolism, persisting fever, heart failure, abscess, and atrioventricular block [10, 14], while SSID 2007 considered only new or progressive heart failure as an indication for a repeated echo [11].

For patients under medical therapy, 3 of the 9 guidelines recommended follow-up echo (class of recommendation: I-IIa, level of evidence: B-C). In addition, follow-up echo is recommended only for complicated IE in SSID 2007 (strength of evidence: II C) and IE with suspected development of complications in AHA 2015 (strength of evidence: I B). Moreover, SSID 2007 stated that TEE is not needed for uncomplicated IE or those with good response to treatment (strength of evidence: II C) [11, 13].

An intraoperative echo examination for IE patients requiring surgery was recommended by 3 of the 9 guidelines (class of recommendation: I, level of evidence: B-C) and was mentioned by BASC 2011 with no formal recommendation [17]. Moreover, BASC/BHRS 2014 mentioned that after IECD removal, patients need a follow-up echo to identify persisting vegetation (level of evidence: C) [15].

At the completion of antibiotic therapy, TEE is recommended by 6 of the 9 guidelines (class of recommendation: I-IIa, level of evidence: C).

**Fig. 4:**
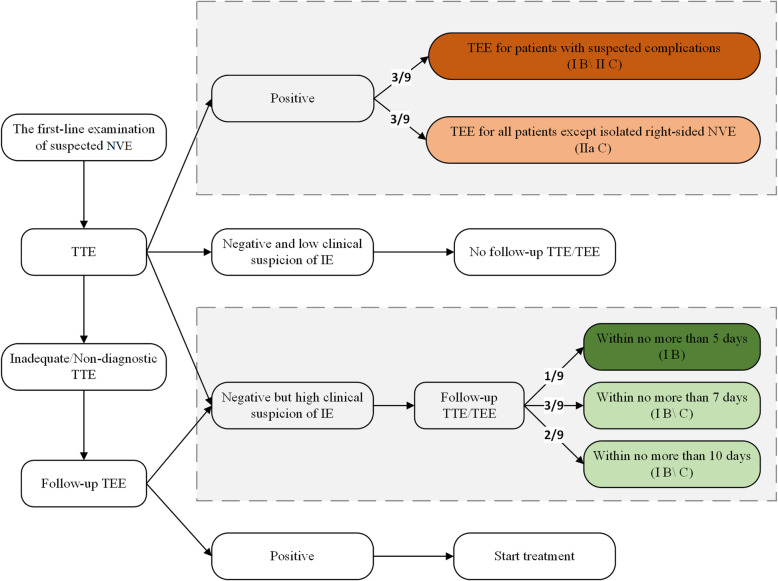
Flowcharts for the controversial clinical scenarios. The dotted box presents the recommendations of the dispute. The ratio marked on the arrow represents the number of guidelines that made the indicated recommendation. The level of recommendation is indicated in brackets. The darker the background color is in the box, the higher the average score of the guidelines that made the recommendation

Regarding whether a follow-up TEE is needed for suspected IE with a positive TTE, the guidelines offered varied guidance based on the features of the target population. For example, BASC 2011 suggested that a positive TTE should be considered to indicate the need for a subsequent TEE, except the isolated right-sided native valve endocarditis (NVE) with good quality TTE examination (no formal recommendation formulated) [17]. ESC 2015 and JCS 2015 excluded unequivocally isolated right-sided native valve IE (class of recommendation: IIa, level of evidence: C) [14, 16]. NHAM 2017 and AHA 2015 advised TEE for patients under concern for complications. NHAM 2017 mentioned that a worsening clinical course and high predisposing risk should be included as indications for TEE (AHA 2015 has a strength of recommendation of I B, and no formal recommendation was given in NHAM 2017) [12, 13]. In addition, SSID 2007 and NHAM 2017 recommended not using TEE after a positive TTE for patients with uncomplicated NVE and low predisposing risk, as well as for patients who had a prompt response to treatment (SSID 2007 has a strength of recommendation of IIIc, and no formal recommendation was given in NHAM 2017) [11, 12]. In summary, as shown in the upper part of Figs. 3, 4 guidelines recommended TEE for all patients except those with isolated right-sided NVE [14, 16, 17], while other 3 guidelines recommended TEE examination only for those with suspected complications (defined differently between the guidelines as showed in Table S5) [11–13].

As shown in the lower part of Fig. 4 and Table S5, when an initial TEE showed a negative result, for patients without a diagnosis of IE but with existing suspicion, 6 of the 9 guidelines recommended a subsequent TEE within a given time limit based on different guidelines (class of recommendation: I, level of evidence: B-C). However, the maximum time limit given by the guidelines varied from 5 to 10 days.

## Discussion

This is the first time that the controversies among recommendations on the appropriate use of echo were evaluated using the AGREE II score of guidelines.

Of the 9 guidelines, the methodology sections of NHAM 2017 and BHRS 2014 indicated that the entire content was compiled based on the AGREE II principle, and both of these guidelines received a score higher than 60% [12, 15]. However, compared with other guidelines that failed to involve certain AGREE II items and thus obtained lower scores, the superior score of NHAM 2017 is largely due to a more general coverage of items rather than a higher grade for each item. The descriptions referring to Item 19 (facilitators and barriers to the application) and Item 20 (the potential resource) in NHAM 2017 were rather perfunctory and lacked in-depth content. Although AGREE II items are recommended during the development of guidelines, comprehensive coverage is just as important as the quality of coverage.

It should also be noted that some studies have shown that exposure to information provided directly by pharmaceutical companies is associated with higher prescribing frequency, higher costs and lower prescribing quality. Therefore, disclosure of potential COI could be necessary [19, 20]. However, it was found that there was no correlation between the proportion of RWI and the AGREE II score involved in the study. This outcome could have been obtained for a variety of reasons, i.e., some guidelines were found to have a high RWI thanks to a more thorough and accomplished disclosure process and less underreporting. For example, ESC has a very detailed COI appendix [14]. In contrast, only NHAM 2017 mentioned that there was no potential COI to disclose and no further detailed relevant content to present. In that case, it cannot be ruled out that the actual RWI proportion of its guideline committee members was concealed and underreported.

Based on the comparison of the recommendations and viewpoints presented by the guidelines, the algorithms for echo use were similar in most cases. Differing recommendations were mainly given on more specific clinical scenarios, and these recommendations were complementary and without conflict. However, there are 2 issues still up for debate.

TTE has been proven to have a sufficient negative predictive value for NVE in patients with low to intermediate risk when strict negative criteria were applied [21, 22]. For these low-risk patients stratified based on clinical judgment with negative TTE, evidence revealed and guidelines proposed that TEE was not necessary [10–14, 16, 17, 23–25]. For a first-line negative TTE with undiagnosed but high suspicion of IE, a follow-up TEE is fully agreed upon. However, the maximum time limits given by the guidelines varies from 5 to 10 days (Fig. 4, Table S5). As TEE may fail to detect abnormalities in the early stage, while the severity of pathology distinguished by echo is helpful for the determination of the following management strategy [26], conducting a follow-up TEE within the appropriate time window could be significant. A recent study has shown that an early (< 4 days) definitive echo is associated with fewer embolic events than echos administered later [27]. Therefore, combining the individual characteristics of each patient, a definitive echocardiogram that identifies the pathology of IE should be timely.

For the most controversial issue of whether a subsequent TEE is mandatory in uncomplicated NVE with initial positive TTE, ESC 2015, JCS 2017 and BASC 2011 recommended TEE for all patients except those with isolated right-sided NVE [14, 16, 17], while NHAM 2017, SSID 2007 and AHA 2015 recommended TEE examination only for those with suspected complications [11–13]. Therefore, for patients (with the exception of those with isolated right-sided NVE) with no or low risk of complications, which are not uncommon in clinical practice, it is unclear whether TEE examination is necessary. On the one hand, TEE is helpful in evaluating the presence of intracardiac complications, offering prognostic information and helping develop treatment plans. On the other hand, if an initial TTE clearly presents vegetation and the probability of complications is low (presented as a small aortic vegetation, mild aortic regurgitation, and normal left ventricular size and function), a subsequent TEE seems to have no incremental value for the treatment strategy [24], which may lead to the overuse of TEE. However, the recommendation that suggested a subsequent TEE after a positive TTE seemed to be less evidence-based and convincing, with a recommendation class of IIa which indicates that weight of evidence and opinion shows usefulness and/or effectiveness, and an evidence level of C which indicates only consensus of opinion. Similarly, the evidence strength of SSID 2007, which explicitly recommended that there was no need to repeat TEE, is C III, which indicates that the evidence supporting the recommendation is weak. Therefore, there is insufficient evidence on this issue, and additional studies with high evidence levels are needed in the future. In addition, isolated right-side NVE was excluded from the adapted population by ESC 2015, JCS 2017 and BASC 2011, which require a subsequent TEE examination as the right-sided structure is located anteriorly and thus closer to the TTE transducer than the transoesophageal transducer, which allows TTE to offer more valuable information. This was also because a subsequent TEE was found to have no additional value for providing new information in isolated right-sided NVE patients [28].

## Conclusion

According to AGREE II and compared with guidelines classified as “Recommended with modification”, those guidelines classified as “Strongly recommended” were more rigorously developed, and the recommendations involved in these guidelines were of higher quality. Clinicians can rely on the guideline quality score when evaluating the recommendations for clinical decision making. In addition, it is also expected that updated to the guidelines will lead to better performance in the domain of “stakeholder involvement”. For the most contentious issue, i.e., whether a subsequent TEE is mandatory in uncomplicated NVE with initial positive TTE, related studies are rarely seen. Therefore, more research on this issue, especially studies with high evidence levels, is desired in the future.

## Supplementary Information

**Additional file 1: Table S1**. Websites of organizations and societies.

**Additional file 2: Table S2**. The detailed search strategy of databases.

**Additional file 3: Table S3**. References of 9 included guidelines.

**Additional file 4: Table S4**. The AGREE II scores of six domains for the included 20 guidelines.

**Additional file 5: Table S5**. Recommendations with controversies.

**Additional file 6: Table S6**. Recommendations without controversies.

## Data Availability

The datasets used and/or analysed during the current study are available from the corresponding author on reasonable request.

## Abbreviations

Echo: Echocardiography
IE: Infective endocarditis
TEE: Transesophageal echocardiography
TTE: Transthoracic echocardiography
AGREE: APPRAISAL OF GUIDELINES FOR RESEARCH & EVALUATION
CSC 2015: Chinese Society of Cardiology, version 2015
SSID 2007: The Swedish Society of Infectious Diseases, version 2007
COI: Conflicts of Interest
RWI: The proportion of panel members with an industry relationship
NHAM 2017: National Heart Association of Malaysia, version 2017
AHA 2015: American Heart Association, version 2015
ESC 2015: European Society of Cardiology, version 2015
BSAC/BHRS 2014: British Society for Antimicrobial Chemotherapy, British Heart Rhythm Society, version 2014
JCS 2017: Japanese Circulation Society, version 2017
BSAC 2011: British Society for Antimicrobial Chemotherapy, version 2011
SEIMC 2015: Spanish Society of Infectious Diseases and Clinical Microbiology, version 2015
ICED: Implantable cardiac electronic device
LCED-LI: Implantable cardiac electronic device lead infection
LCED-IE: Implantable cardiac electronic device associated native or prosthetic valve endocarditis
NVE: Native valve endocarditis
