# Supplementary material for: An appraisal of clinical practice guidelines for the appropriate use of echocardiography for adult infective endocarditis—the timing and mode of assessment (TTE or TEE)
Source: BMC Infect Dis. 2021 Jan 21;21:92. doi: 10.1186/s12879-021-05785-6 (PMC7819184; doi:10.1186/s12879-021-05785-6)
Supplement: Supplementary file 1 — Additional file 1: Table S1. Websites of organizations and societies. [file 12879_2021_5785_MOESM1_ESM.docx]

Table S1
Websites of organizations and societies

| organizations and societies | Websites |
| --- | --- |
| The Alliance for the Implementation of Clinical Practice Guidelines | https://aicpg.org/ |
| Guideline Central Library | https://www.guidelinecentral.com/summaries/ |
| Guidelines International Network. | https://g-i-n.net/home |
| Johns Hopkins Guides | https://www.hopkinsguides.com/hopkins/ |
| The Japanese Association for infectious disease | http://www.kansensho.or.jp/ref/index.html |
| American college of cardiology | https://www.acc.org/ |
| American Heart Association | https://www.heart.org/ |
| European Society of Cardiology | https://www.escardio.org/ |
| Canadian Cardiovascular Society | https://www.ccs.ca/en/ |
| National Institute for Health and Care Excellence | https://www.nice.org.uk/ |
| Healthcare Improvement Scotland | https://www.sign.ac.uk/ |
| Russian society of cardiology | https://scardio.ru/en/ |
| Taiwan society of cardiology | http://www.tsoc.org.tw/ |
| Korean society of cardiology | https://www.circulation.or.kr:4443/eng/ |
| The Cardiac Society of Australia and New Zealand | https://www.csanz.edu.au/ |
| Egyptian society of cardiology | http://egsc.org.eg/#/main/home |
| French society of cardiology | https://www.sfcardio.fr/ |
| the National Heart Association of Malaysia | https://www.malaysianheart.org/ |
| Japanese Circulation society | http://www.j-circ.or.jp/english/ |
