# Supplementary material for: An appraisal of clinical practice guidelines for the appropriate use of echocardiography for adult infective endocarditis—the timing and mode of assessment (TTE or TEE)
Source: BMC Infect Dis. 2021 Jan 21;21:92. doi: 10.1186/s12879-021-05785-6 (PMC7819184; doi:10.1186/s12879-021-05785-6)
Supplement: Supplementary file 2 — Additional file 2: Table S2. The detailed search strategy of databases. [file 12879_2021_5785_MOESM2_ESM.docx]

Table S2
The detailed search strategy of databases

| Pubmed | | | |
| --- | --- | --- | --- |
| #1 | | infective endocarditis OR infectious endocarditis OR endocarditis | |
| #2 | | guideline | |
| #3 | | #1 AND #2 | |
| #4 | | ("2000/01/01"[Date - Publication]: "3000" [Date - Publication])) AND English[Language] | |
| #5 | | #3 AND #4 | |
| Web of science | | | |
| #1 | | TS= infective endocarditis OR TS=infectious endocarditis OR TS=endocarditis | |
| #2 | | TI=guideline | |
| #3 | | #1 AND #2 | |
| #4 | | #3 AND ‘practice guideline’: ti,ab,kw | |
| #5 | | #4 AND #2 | |
| #6 | | #3 OR #5 | |
| #7 | | #6 AND PY=2000-2020 | |
| EMBASE | | |  |
| #1 | | infective endocarditis OR infectious endocarditis OR endocarditis |  |

| #2 | practice guideline’: ti,ab,kw |
| --- | --- |
| #3 | #1 AND #2 |
| #4 | English: la AND [2000-2020]/py |
| #5 | #3 AND #4 |
