# Supplementary material for: An appraisal of clinical practice guidelines for the appropriate use of echocardiography for adult infective endocarditis—the timing and mode of assessment (TTE or TEE)
Source: BMC Infect Dis. 2021 Jan 21;21:92. doi: 10.1186/s12879-021-05785-6 (PMC7819184; doi:10.1186/s12879-021-05785-6)
Supplement: Supplementary file 3 — Additional file 3: Table S3. References of 9 included guidelines. [file 12879_2021_5785_MOESM3_ESM.docx]

Table S3
References of 9 included guidelines.

| Guidelines identifier, Year | References |
| --- | --- |
| NHAM 2017 | National Heart Association of Malaysia, Academy of Medicine Malaysia. Clinical practice guidelines for the prevention, diagnosis & management of infective endocarditis. Putrajaya: Clinical Practice Guidelines (CPG) Secretariat; 2016. |
| AHA 2015 | Baddour LM, Wilson WR, Bayer AS, Fowler VG, Jr., Tleyjeh IM, Rybak MJ, Barsic B, Lockhart PB, Gewitz MH, Levison ME, Bolger AF, Steckelberg JM, Baltimore RS, Fink AM, O'Gara P, Taubert KA. Infective endocarditis in adults: Diagnosis, antimicrobial therapy, and management of complications: A scientific statement for healthcare professionals from the American Heart Association. Circulation 2015;132(15):1435-1486. |
| ESC 2015 | Habib G, Lancellotti P, Antunes MJ, Bongiorni MG, Casalta JP, Del Zotti F, Dulgheru R, El Khoury G, Erba PA, Iung B, Miro JM, Mulder BJ, Plonska-Gosciniak E, Price S, Roos-Hesselink J, Snygg-Martin U, Thuny F, Tornos Mas P, Vilacosta I, Zamorano JL. 2015 ESC guidelines for the management of infective endocarditis: The task force for the management of infective endocarditis of the European Society of Cardiology (ESC). endorsed by: European Association for Cardio-Thoracic Surgery (EACTS), the European Association of Nuclear Medicine (EANM). Eur Heart J 2015;36(44):3075-3128. |
| BSAC/BHRS 2014 | Sandoe JA, Barlow G, Chambers JB, Gammage M, Guleri A, Howard P, Olson E, Perry JD, Prendergast BD, Spry MJ, Steeds RP, Tayebjee MH, Watkin R. Guidelines for the diagnosis, prevention and management of implantable cardiac electronic device infection. Report of a joint Working Party project on behalf of the British Society for Antimicrobial Chemotherapy (BSAC, host organization), British Heart Rhythm Society (BHRS), British Cardiovascular Society (BCS), British Heart Valve Society (BHVS) and British Society for Echocardiography (BSE). J Antimicrob Chemother 2015;70(2):325-359. |
| JCS 2017 | Nakatani S, Ohara T, Ashihara K, Izumi C, Iwanaga S, Eishi K, Okita Y, Daimon M, Kimura T, Toyoda K, Nakase H, Nakano K, Higashi M, Mitsutake K, Murakami T, Yasukochi S, Okazaki S, Sakamoto H, Tanaka H, Nakagawa I, Nomura R, Fujiu K, Miura T, Morizane T. JCS 2017 guideline on prevention and treatment of infective endocarditis. Circ J 2019;83(8):1767-1809. |
| BSAC 2011 | Gould FK, Denning DW, Elliott TS, Foweraker J, Perry JD, Prendergast BD, Sandoe JA, Spry MJ, Watkin RW, Working Party of the British Society for Antimicrobial C. Guidelines for the diagnosis and antibiotic treatment of endocarditis in adults: a report of the Working Party of the British Society for Antimicrobial Chemotherapy. J Antimicrob Chemother 2012;67(2):269-289. |
| SEIMC 2015 | Gudiol F, Aguado JM, Almirante B, Bouza E, Cercenado E, Dominguez MA, Gasch O, Lora-Tamayo J, Miro JM, Palomar M, Pascual A, Pericas JM, Pujol M, Rodriguez-Bano J, Shaw E, Soriano A, Valles J. Diagnosis and treatment of bacteremia and endocarditis due to Staphylococcus aureus. A clinical guideline from the Spanish Society of Clinical Microbiology and Infectious Diseases (SEIMC). Enferm Infecc Microbiol Clin 2015;33(9):625.e621-625.e623. |
| CSC 2015 | Dong YG, Huang J, Li GH, Li LW, Li WM, Li XL, Liu XH, Liu ZY, Lu YX, Ma AQ, Sun HS, Wang H, Wen XH, Xu DJ, Yang JF, Zhang J, Zhao H, Zhou JM, Zhu LP (2015) Guidelines for the prevention, diagnosis, and treatment of infective endocarditis in adults: The Task Force for the Prevention, Diagnosis, and Treatment of Infective Endocarditis in Adults of Chinese Society of Cardiology of Chinese Medical Association, and of the Editorial Board of Chinese Journal of Cardiology. Eur Heart J Suppl; 2015: 17 (Suppl C): C1-C16. doi: 10.1093/eurheartj/suv031 |
| SSID 2007 | Westling K, Aufwerber E, Ekdahl C, Friman G, Gardlund B, Julander I, Olaison L, Olesund C, Rundstrom H, Snygg-Martin U, Thalme A, Werner M, Hogevik H. Swedish guidelines for diagnosis and treatment of infective endocarditis. Scand J Infect Dis 2007;39(11-12):929-946. |
