# Supplementary material for: An appraisal of clinical practice guidelines for the appropriate use of echocardiography for adult infective endocarditis—the timing and mode of assessment (TTE or TEE)
Source: BMC Infect Dis. 2021 Jan 21;21:92. doi: 10.1186/s12879-021-05785-6 (PMC7819184; doi:10.1186/s12879-021-05785-6)
Supplement: Supplementary file 4 — Additional file 4: Table S4. The AGREE II scores of six domains for the included 20 guidelines. [file 12879_2021_5785_MOESM4_ESM.docx]

Table S4

The AGREE II scores of six domains for the included 20 guidelines.

| Guidelines identifier, Year‡ | Scope and purpose | Stakeholder involvement | Rigour of  Development | Clarity of presentation | Application | Editorial independence |
| --- | --- | --- | --- | --- | --- | --- |
| AHA, 2015 | 62% | 28% | 88% | 94% | 79% | 100% |
| BSAC, 2011 | 83% | 22% | 40% | 100% | 38% | 75% |
| BSAC/BHRS, 2014 | 89% | 83% | 46% | 72% | 42% | 83% |
| CSC,2015 | 62% | 22% | 35% | 89% | 25% | 0% |
| ESC, 2015 | 67% | 33% | 85% | 94% | 67% | 100% |
| JCS,2017 | 78% | 56% | 44% | 89% | 42% | 42% |
| NHAM, 2017 | 94% | 62% | 75% | 89% | 83% | 75% |
| SEIMC, 2015 | 72% | 22% | 58% | 67% | 38% | 58% |
| SSID, 2017 | 56% | 11% | 33% | 89% | 21% | 0% |

‡ The guideline references were listed in Table S5.
