# Supplementary material for: An appraisal of clinical practice guidelines for the appropriate use of echocardiography for adult infective endocarditis—the timing and mode of assessment (TTE or TEE)
Source: BMC Infect Dis. 2021 Jan 21;21:92. doi: 10.1186/s12879-021-05785-6 (PMC7819184; doi:10.1186/s12879-021-05785-6)
Supplement: Supplementary file 5 — Additional file 5: Table S5. Recommendations with controversies. [file 12879_2021_5785_MOESM5_ESM.docx]

Table S5
Recommendations with controversies

| Clinical Scenarios | Guidelines identifier, Year‡ | Specific subgroups features | Mode of echocardiography | | | | | | Comments | Strength of recommendations¶ |
| --- | --- | --- | --- | --- | --- | --- | --- | --- | --- | --- |
|  |  |  | TTE | | | TEE | | |  |  |
| Remained high clinical suspicion of IE despite negative initial TTE and TEE examinations | NHAM, 2017 | — | TTE | and/or | | | | TEE | Within 7 days if clinical findings changed. | I C |
|  | AHA, 2015 | Patients with an initial negative TEE. | — | | | | TEE | | Within in 3 to 5 days or sooner if clinical findings change. | I B |
|  | ESC, 2015 | — | TTE | and/or | | | | TTE | Within 5 to 7 days. | I B |
|  | JCS, 2017 | — | Repeated echo is recommended. | | | | | | After 3 to 7 days. | I C |
|  | BSAC, 2011 | — | TTE | or | | | | TTE | Within 7 to 10 days. | C |
|  | CSC, 2015 | — | TTE | or | | | | TTE | Within 7 to 10 days. | I B |
| Suspected IE with positive TTE (whether a follow-up TEE is needed) | NHAM, 2017 | Worsening clinical course/high predisposing risk/echo suggests possible complications. | — | | | | Recommend | | No formal recommendation come with level of evidence. | — |
|  | AHA, 2015 | Patients with concern for intracardiac complications. | — | | | | Recommend | | — | I B |
|  | ESC, 2015 | All patients expect isolated right-sided native valve IE with unequivocal TTE finding. | — | | | | Recommend | | To rule out local complications. | IIa C |
|  | JCS, 2017 | All patients expect isolated right cardiac valve IE. | — | | | | Recommend | | — | IIa C |
|  | BASC, 2011 | All adults with a positive TTE expect isolated right-sided native valve IE. | — | | Recommend | | | | No formal recommendation attached with level of evidence. | — |
|  | SSID, 2007 | Uncomplicated native valve IE and prompt response to treatment. | — | | | | No follow-up TEE | | — | III C |

‡ The guideline references were listed in Table S4; ¶ The level of evidence on each recommendation was adopted from respective guideline; Echo: echocardiography; TEE: transesophageal echocardiography; TTE: transthoracic echocardiography.
