# Supplementary material for: An appraisal of clinical practice guidelines for the appropriate use of echocardiography for adult infective endocarditis—the timing and mode of assessment (TTE or TEE)
Source: BMC Infect Dis. 2021 Jan 21;21:92. doi: 10.1186/s12879-021-05785-6 (PMC7819184; doi:10.1186/s12879-021-05785-6)
Supplement: Supplementary file 6 — Additional file 6: Table S6. Recommendations without controversies. [file 12879_2021_5785_MOESM6_ESM.docx]

Table S6
Recommendations without controversies

| Clinical Scenarios | Guidelines identifier, Year‡ | Specific subgroups features | Mode of echocardiography | | | | | | Comments | Strength of recommendations¶ |
| --- | --- | --- | --- | --- | --- | --- | --- | --- | --- | --- |
|  |  |  | TTE | | | TEE | | |  |  |
| The first-line modality of suspected IE | NHAM, 2017 | — | Echo is recommended with TTE being the first line imaging investigation. | | | | | | As TTE is non-invasive and widely available;  TEE should be performed subsequently if indicated. | I B |
|  | AHA, 2015 | — | Recommend | | | — | | | — | I B |
|  | ESC, 2015 | — | Recommend | | | — | | | — | I B |
|  | JSC, 2017 | — | Recommend | | | — | | | — | I B |
|  | BSAC, 2011 | — | Recommend | | | — | | | Which should be done as soon as possible, ideally within 24h. | C |
|  | CSC, 2015 | — | Recommend | | | — | | | — | I B |
|  | SSID, 2007 | — | Echo is recommended with TEE being the first choice. | | | | | | Because of its higher sensitivity both regarding vegetations and complications as aortic root abscesses. | I B |
| Suspected IE with non-diagnostic/inadequate TTE examination | NHAM, 2017 | — | — | | | Recommend | | | — | I C |
|  | AHA, 2015 | — | — | | | Recommend | | | — | I B |
|  | ESC, 2015 | — | — | | | Recommend | | | — | I B |
|  | JSC, 2017 | — | — | | | Recommend | | | — | I B |
|  | CSC, 2015 | — | — | | | Recommend | | | — | I B |
| Suspected IE with a prosthetic heart valve/intracardiac device | NHAM, 2017 | — | — | | | Recommend | | | — | I B |
|  | ESC, 2015 | — | — | | | Recommend | | | — | I B |
|  | BSAC/BHRS, 2014 | i) Patients with clinical suspected LCED-LI or LCED-IE infection. | Echo is recommended | | | | | | — | B |
|  |  | ii) Patients with diagnosed ICED infection. | Echo is recommended | | | | | | Within 24 hours. | C |
|  |  | iii) Patients with suspected generator pocket infection concurrent ICED-LI or ICED-IE. | Echo is recommended | | | | | | — | C |
|  | JCS, 2017 | — | — | | | Recommend | | | — | I B |
|  | SSID, 2007 | — | — | | | Recommend | | | — | I B |
| In patients with SAB bacteremia | AHA, 2015 | — | An early TTE should be performed if TEE is not immediately available, then a further TEE is needed. | | | | | | No formal recommendation come with level of evidence. | — |
|  | ESC, 2015 | — | Echo is recommended. | | | | | | TTE or TEE should be considered according to individual patient risk factors and the mode of acquisition of SAB. | IIa B |
|  | BSAC/BHRS,  2014 | Patients with an ICED and SAB in one or more blood cultures. | Echo is recommended | | | | | | or other microorganisms in multiple blood cultures. | B |
|  | JCS, 2017 | — | Echo is recommended. | | | | | | Since IE is highly likely in the cases of SAB. | IIa B |
|  | BSAC, 2011 | — | Echo is recommended. | | | | | | Ideally within the first week of treatment or within 24 h if there is other evidence to suggest IE | B |
|  | SEIMC, 2015 | i) Patients with complicated SAB. | Echo is suggested. | | | | | | No formal recommendations. | — |
|  |  | ii) Cather-related bacteremia by SAB. | — | | | | Recommend | | No formal recommendations. | — |
|  | SSID, 2007 | — | — | | | | Recommend | | — | I C |
| Onset of suspected complications of IE | ESC, 2015 | — | Repeated TTE | and/or | | | | Repeated TTE | As soon as a new complication of IE is suspected (new murmur, embolism, persisting fever, HF, abscess, atrioventricular block) | I B |
|  | JCS, 2017 | — | Follow-up Echo is recommended | | | | | | After the onset of new complications. | I B |
|  | BASC, 2011 | — | Follow-up TTE | and/or | | | | Follow-up TEE | If there is evidence of cardiac complication. | B |
|  | SSID, 2007 | — | Repeated TTE | and/or | | | | Repeated TTE | If patients develop a new or progressive heart failure. | I C |
|  | CSC, 2015 | — | Repeated TTE | and | | | | Repeated TTE | As soon as a new complication of IE is suspected (new murmur, embolism, persisting fever, heart failure, and atrioventricular block). | I B |
| IE requiring surgery | NHAM, 2017 | — | Intraoperative Echo is recommended. | | | | | | — | I B |
|  | ESC, 2015 | — | Intraoperative Echo is recommended. | | | | | | — | I B |
|  | BASC/BHRS, 2014 | Patients after IECD removal. | Repeated Echo is recommended | | | | | | To identify persisting valve or mural vegetations. | C |
|  | BCSC, 2011 | — | Intraoperative Echo is recommended. | | | | | | No formal recommendation. | — |
|  | CSC, 2015 | — | Intraoperative Echo is recommended. | | | | | | — | I C |
| Follow-up Echo under medical therapy | NHAM, 2017 | Low predisposing risk/good response to treatment. | Repeated TTE. | | — | | | | No formal recommendation. | — |
|  | AHA, 2015 | when clinical features suggest a new development of intracardiac complications. | — | | Repeated TEE. | | | | — | I B |
|  | ESC, 2015 | Uncomplicated IE. | Repeated TTE | and/or | | | | Repeated TTE | To detect new silent complications and monitor vegetation size. The timing and mode (TTE or TEE) depend on the initial findings, type of microorganism, and initial response to therapy. | IIa B |
|  | JCS, 2017 | — | i) Follow-up Echo is recommended | | | | | | For evaluating therapeutic effect. | I C |
|  |  | — | ii) Follow-up Echo is recommended | | | | | | For evaluating the onset of asymptomatic intracardiac complications. | IIa B |
|  | BSAC, 2011 | — | i) Routine repeated Echo is not recommended. | | | | | | — | C |
|  |  | — | ii) Repeated TTE | and/or | | | | Repeated TTE | For suboptimal response to treatment. | B |
|  | SSID, 2007 | Complicated IE. | i) Repeated TTE | and/or | | | | Repeated TTE | — | II C |
|  |  | for uncomplicated IE and prompt response to treatment | ii) — | | | | No repeated TEE | | — | II C |
|  | CSC, 2015 | — | Repeated TTE | and | | | | Repeated TTE | To detect new silent complications and to monitor vegetation size. The timing and mode (TTE or TEE) depend on the initial findings, type of microorganism, and initial response to therapy. | IIa B |
| At completion of antibiotic therapy | NHAM, 2017 | — | Recommend | | | | — | | To record baseline features. | IIa C |
|  | AHA, 2015 | — | Recommend | | | | — | | To establish baseline features. | IIa C |
|  | ESC, 2015 | — | Recommend | | | | — | | For evaluation of cardiac and valve morphology and function. | I C |
|  | JCS, 2017 | — | Recommend | | | | — | | For evaluation of effects of antibiotics. | I C |
|  | BSAC, 2011 | — | Recommend | | | | — | | For evaluation of cardiac and valve morphology and function. | C |
|  | CSC, 2015 | — | Recommend | | | | — | | For evaluation of cardiac and valve morphology and function. | I C |

‡ The guideline references were listed in Table S4; ¶ The level of evidence on each recommendation was adopted from respective guideline; Echo: echocardiography; TEE: transesophageal echocardiography; TTE: transthoracic echocardiography; ICED: implantable cardiac electronic device; ICED-LI: implantable cardiac electronic device lead infection; ICED-IE: implantable cardiac electronic device associated native or prosthetic valve endocarditis; SAB: S.aureus bacteremia.
